# Vector sequence contamination of the *Plasmodium vivax* sequence database in PlasmoDB and *In silico* correction of 26 parasite sequences

**DOI:** 10.1186/s13071-015-0927-x

**Published:** 2015-06-12

**Authors:** Zhi-Yong Tao, Xu Sui, Cao Jun, Richard Culleton, Qiang Fang, Hui Xia, Qi Gao

**Affiliations:** Department of Parasitology, Bengbu Medical College, Bengbu, People’s Republic of China; Jiangsu Institute of Parasitic Diseases, Wuxi, China; Key Laboratory of Parasitic Disease Control and Prevention, Ministry of Health, Wuxi, China; Jiangsu Provincial Key Laboratory of Parasite Molecular Biology, Wuxi, China; Malaria Unit, Institute of Tropical Medicine, Nagasaki University, Sakamoto, Nagasaki Japan

**Keywords:** PlasmoDB, *Plasmodium vivax*, Cloning vector, Genome

## Abstract

We found a 47 aa protein sequence that occurs 17 times in the *Plasmodium vivax* nucleotide database published on PlasmoDB. Coding sequence analysis showed multiple restriction enzyme sites within the 141 bp nucleotide sequence, and a His6 tag attached to the 3’ end, suggesting cloning vector origins. Sequences with vector contamination were submitted to NCBI, and BLASTN was used to cross-examine whole-genome shotgun contigs (WGS) from four recently deposited *P. vivax* whole genome sequencing projects. There are at least 26 genes listed in the PlasmoDB database that incorporate this cloning vector sequence into their predicted provisional protein products.

## Findings

Genome databases are of great value for biomedical research, and have significantly advanced our understanding of the biology of multiple parasite species, including *Plasmodium falciparum* and *Plasmodium vivax*, the two most common malaria parasites [1, 2]. The latter genome sequence was produced by shotgun sequencing by Carlton *et al.* at TIGR in 2008 at five fold coverage, and is deposited at GenBank and PlasmoDB [3]. Assembly errors are inevitable when constructing genomes, and, in the case of intracellular parasites, contamination with host DNA sequence also poses a problem. Indeed, recent research has shown that many published genomes, including mammalian, contain contaminating sequence from a variety of microorganisms [4]. Considering gene prediction errors and malaria parasites specifically, Lu *et al.* reported that about 20 % of genes are incorrectly predicted in the *P. falciparum* genome database, although these are mostly due to errors arising from the gene prediction software used [5].

**Fig. 1:**
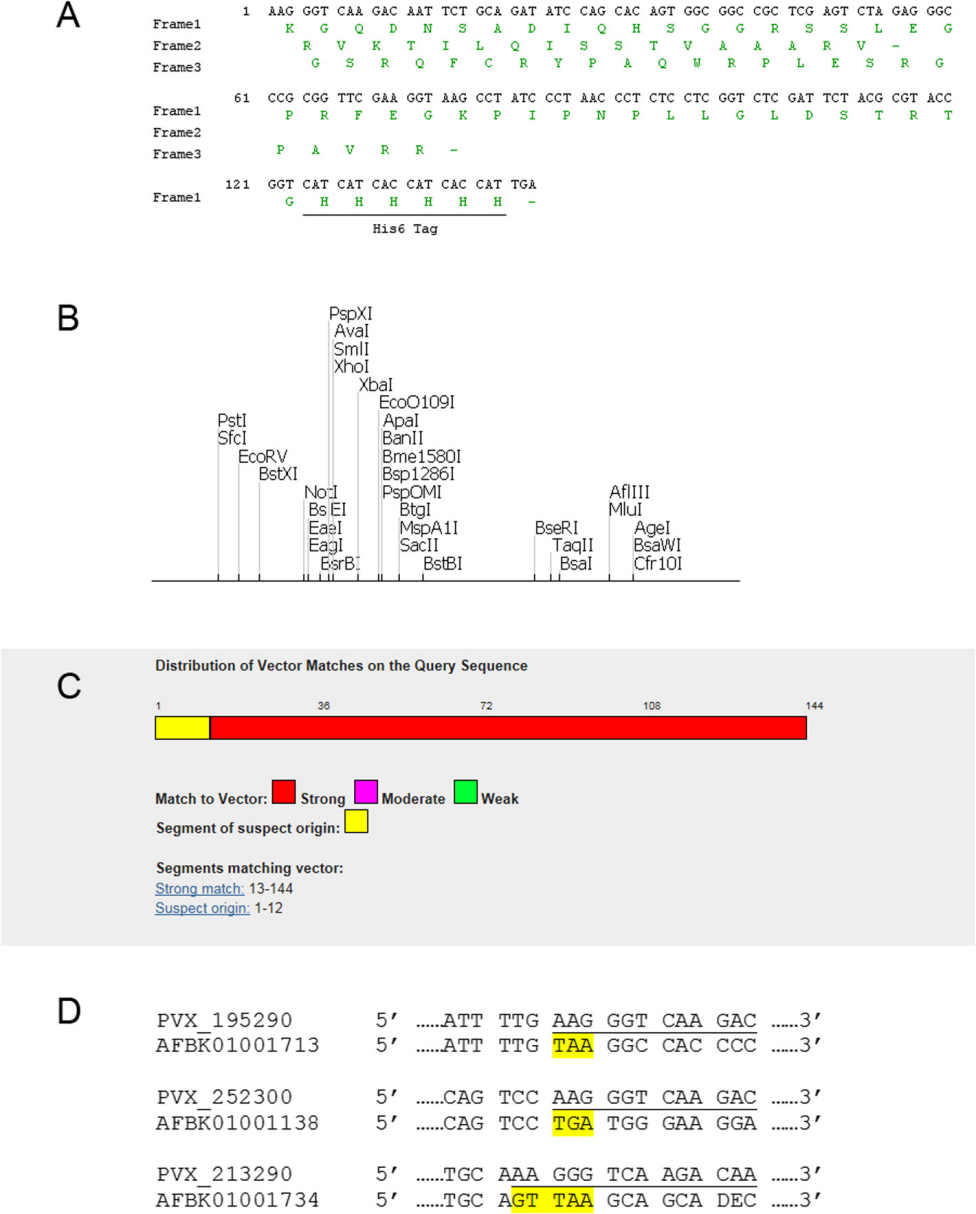
Cloning vector source sequence contamination in PlasmoDB. **a**: A 141 bp vector source sequence with a his6 tag repeatedly occurred in the *Plasmodium vivax* nucleotide database. **b**: Dozens of restriction enzyme sites are present in the sequence. **c**: VecScreen search showed the contaminating sequence strongly match to pMQ354. **d**: Typical errors in Sal-1 strain sequencing results due to the contaminating sequence. The missing ends are marked in yellow, and contaminating vector sequences are underlined

During a search for repetitive protein fragments in the *P. vivax* genome conducted on the nucleotide sequences deposited in PlasmoDB [6] we found a 47 amino acid (aa) sequence (KGQDNSADIQHSGGRSSLEGPRFEGKPIPNPLLGLDSTRTGHHHHHH) repeated a total of 17 times in several annotated contigs. A His6 tag (Fig 1A) was attached to the 3’ end, and multiple restriction enzyme sites (Fig 1B) were present within the 141 bp nucleotide sequence (AAG GGT CAA GAC AAT TCT GCA GAT ATC CAG CAC AGT GGC GGC CGC TCG AGT CTA GAG GGC CCG CGG TTC GAA GGT AAG CCT ATC CCT AAC CCT CTC CTC GGT CTC GAT TCT ACG CGT ACC GGT CAT CAT CAC CAT CAC CAT). This sequence, when run through a VecScreen search (NCBI, http://www.ncbi.nlm.nih.gov/tools/vecscreen/) shows significant similarity to the promoter probe vector pMQ354 (Fig 1C). These features suggest cloning vector sequence contamination. We performed BLASTN searches of these 17 coding sequences against whole-genome shotgun contigs (WGS) of four whole genome sequences (India VII [GenBank: AFMK01000000], North Korean [GenBank: AFBK01000000], Brazil I [GenBank: AFNI01000000], Mauritania I [GenBank: AFNJ01000000]) [7]. All hits were aligned with the reference sequence, and the results showed missing or substituted base pairs at the 3′ end of the query sequences, resulting in the absence of the correct stop codon of the parasite gene, and the incorporation of the vector sequence into the predicted parasite gene protein product, which then terminated at the vector stop codon. Considering that there may be a possibility of frame shifting, we translated the coding sequence in all three frames (Fig 1A), and frames two and three protein were used as query sequences against the PlasmoDB protein database. This resulted in five and four sequence hits respectively, and these nine sequences were subjected to alignment and correction as described before. In total, we discovered 26 sequences in PlasmoDB contaminated by the vector sequence (Table 1).

**Table 1:** Correction of 26 genes affected by a contaminated cloning vector sequence in PlasmoDB

ID	PlasmoDB ID	GenBank accession number	Length (bp)	
			Before correction	After correction
1	PVX_253300	XM_001612328	1,086	945
2	PVX_250300	XM_001612323	1,047	906
3	PVX_211290^a^	XM_001612311	945	807
4	PVX_226290^a^	XM_001612298	792	741
5	PVX_214290^a^	XM_001612318	861	792
6	PVX_215290^a^	XM_001612317	861	793
7	PVX_220290	XM_001612333	654	513
8	PVX_252300	XM_001612332	1,149	1,008
9	PVX_222290^b^	XM_001612349	1,233	1,098
10	PVX_196290^b^	XM_001612337	1,173	1,101
11	PVX_195290	XM_001612373	1,893	1,752
12	PVX_231290	XM_001612334	639	498
13	PVX_213290	XM_001612274	513	441
14	PVX_249300	XM_001612331	1,113	972
15	PVX_227290	XM_001612370	1,902	1,761
16	PVX_240290^c^	XM_001612308	942	801
17	PVX_235290^c^	XM_001612320	717	576
18	PVX_254300	XM_001612327	1,062	921
19	PVX_200290^d^	XM_001612305	921	876
20	PVX_201290^d^	XM_001612303	828	780
21	PVX_206290^d^	XM_001612319	924	876
22	PVX_208290^d^	XM_001612329	1,017	876
23	PVX_216290^e^	XM_001612279	711	570
24	PVX_218290^e^	XM_001612281	711	570
25	PVX_237290^e^	XM_001612314	711	570
26	PVX_217290^e^	XM_001612282	621	570

^a, b, c, d,^ ^e^:Represent duplicated sequences respectively

Generally, cloning vector source sequences are relatively easily recognized by a variety of tools, such as VecScreen. The *P. vivax* database has been updated more than ten times [8], and yet this vector sequence contamination persists, suggesting that it may have special characteristics that render it difficult to identify automatically. Attempted PCR amplification of Sal-1 genomic DNA using primers specific for the potential contaminating sequence would provide definitive proof of whether these sequences really are present in the genome, a scenario we believe to be highly unlikely.

The publication of four geographical reference strain whole genome sequences now provides an opportunity for the correction of the genome sequence of the Sal-I reference genome. Given our findings, it is possible that further interrogation of the *P. vivax* genome deposited in PlasmoDB may reveal further contamination. It is also possible that any previous work that made use of these sequences may require reappraisal.
